# Platinum Growing Scarce

**Published:** 1889-07

**Authors:** 


### PLATINUM GROWING SCARCE.

The increased demand for platinum during the past few years has tended to raise the price very rapidly, and the last eighteen months has seen a rise of two dollars an ounce. It has doubled in value in the last decade, with the consumption increasing; and, with no new fields being discovered, but one natural result can follow, and that is, a very marked further increase in price in the near future. The increased demand is not so much due to its enormous consumption in the manufacture of teeth as to its adoption in the electric-lighting process,—every glass globe containing a coil of platinum wire.

Platinum is the only metal thus far known that can be used for pins in the manufacture of porcelain teeth; the source of supply is confined almost entirely to the Ural Mountains, in Russia; some little has been discovered in South America, but none worth speaking of. Considerable money has been spent prospecting for mines wherever indication seemed to point to the discovery of the ore, but so far without any tangible results. In addition to the scarcity of the article, the entire product of the world is in the hands of a powerful European syndicate, which manipulates the market at will and controls prices.

The increase in the price of the metal can have only one result, and that is to raise the price of teeth or drive the manufacturer out of business. This specially relates to the so-called cheap teeth, which have during the past few years been so much improved, some grades having reached that state of perfection that the price can easily be advanced and yet compete with the higher-priced teeth; but other manufacturers will no doubt be forced to retire from business. Unless some means is found to substitute other metals or methods of manufacture, we very much fear that the dollar tooth will become a thing of the past.

